# Supplementary figures and images for: Epistasis and cryptic QTL identified using modified bulk segregant analysis of copper resistance in budding yeast
Source: bioRxiv. 2024 Nov 12:2024.10.28.620582. Preprint. [Version 3] doi: 10.1101/2024.10.28.620582 (PMC11601411; doi:10.1101/2024.10.28.620582)

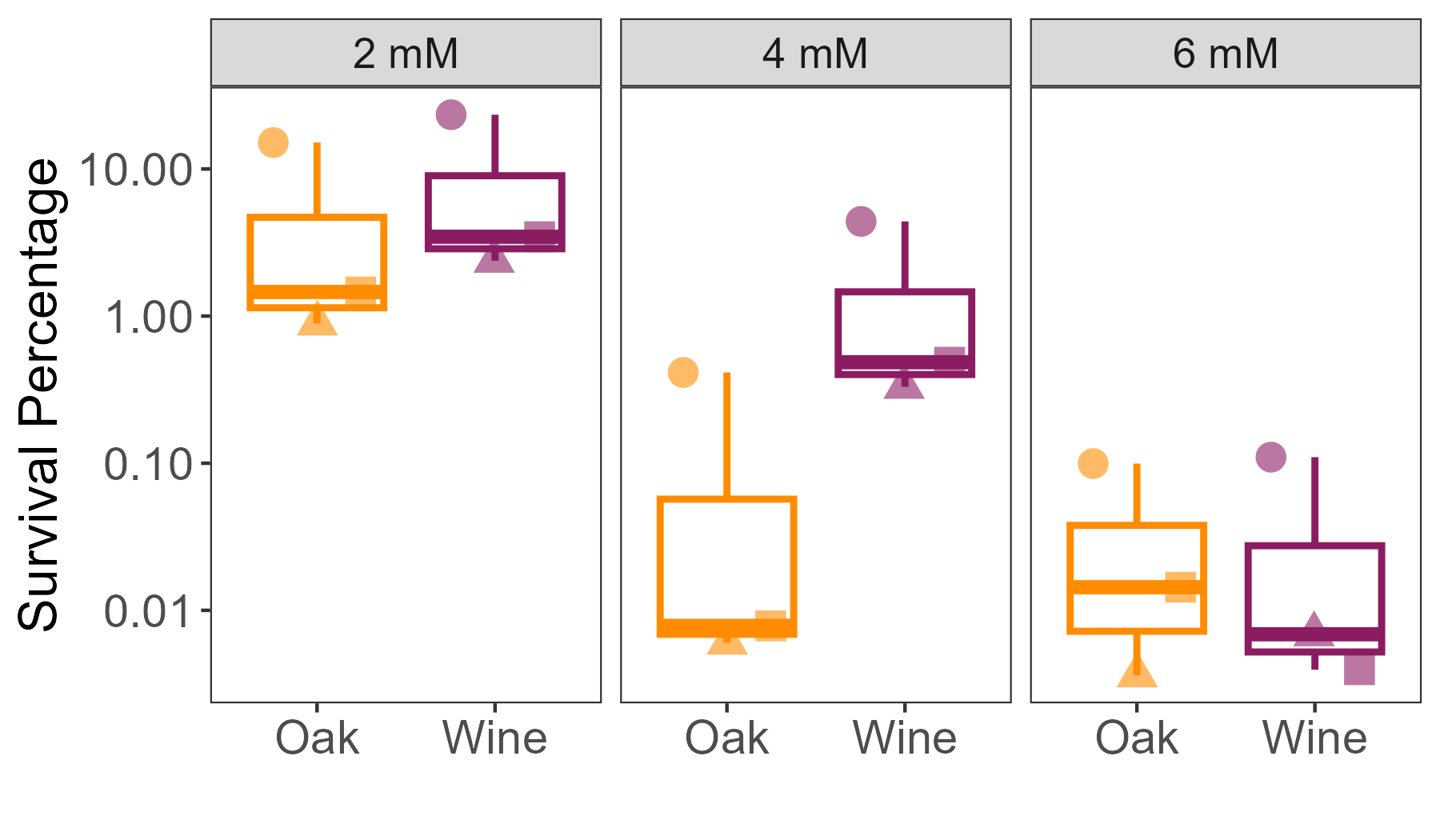

Supplement: Supplement 1 — S1 Fig. Survival percentage by strain in CuSO4. Boxplots of survival percentages of Oak (NCYC3631) and Wine (NCYC3591) haploid strains at different concentrations of copper sulfate are shown. Replicate experiments carried out on different dates are represented by different shapes. [file media-1.tif]

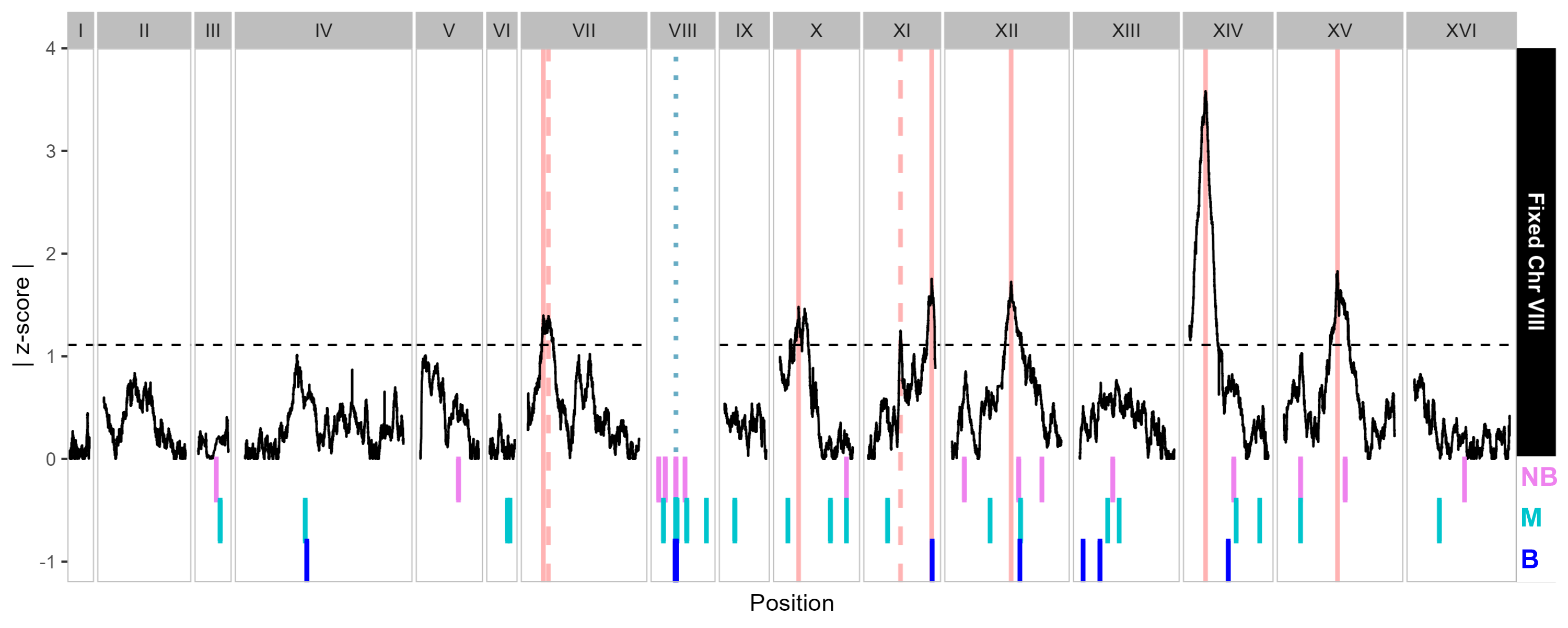

Supplement: Supplement 2 — S2 Fig. Comparison with copper sulfate interaction peaks from previous studies. Interactions with chromosome VIII identified in our study (shown in black trace of the absolute z-score by genome position) are compared with interaction QTL involving chromosome VIII identified in other copper-resistance studies, which used both different experimental designs and different strains. Beneath the trace from this study, NB (magenta) indicates interaction QTL as identified by Nguyen Ba et al [21], M (turquoise) represents interaction hotspots between strains from Matsui et al. [22], and B (dark blue) represents interaction QTL as identified by Bloom et al [4]. Red vertical lines indicate significant peaks called in our study. CUP1 is indicated by the vertical light blue dotted line on Chromosome VIII. [file media-2.tif]
